# Differences in proximal and intimacy-related defense mechanisms among patients with cancer in different psychological stages of dying

**DOI:** 10.3389/fpsyg.2024.1329043

**Published:** 2024-02-20

**Authors:** Jia Zhou, Mengxiang Li, Jiarui Dong, Hui Shi, Meihong Shi

**Affiliations:** ^1^School of Humanities and Management Science, Southwest Medical University, Luzhou, China; ^2^School of Nursing, Southwest Medical University, Luzhou, China

**Keywords:** psychological stages of dying, cancer, proximal defense, intimacy, death anxiety thought accessibility test, self-control questionnaire, rumination reflection questionnaire, attachment type test

## Abstract

**Purpose:**

This study measured three of the psychological stages of dying in patients with cancer and explored the differences in proximal and intimacy-related defense mechanisms at each stage.

**Patients and methods:**

A total of 220 cancer patients were recruited for this study; 168 patients met the inclusion criteria and were included in the data analysis. The participants were divided into three groups using the “Death Attitudes Questionnaire Revised” (1994) and then completed the Death-Thought Accessibility Test, Self-Control Questionnaire, Rumination Reflection Questionnaire, Attachment Type Test, Intimacy Test, External Control Test, and Positive and Negative Affect Scale.

**Results:**

In the death avoidance stage, which represents a defense stage without cognitive processes, patients are in an irrational state with the highest level of self-control and the lowest level of external control; they tend to prefer close relationships with many people while experiencing high levels of fear and depression. In the bargaining stage, which represents a biased cognitive defense stage, the level of rationality increases, the level of fear and depression decreases, and patients tend to prefer relationships with many people that do not involve intimacy. In the neutral death acceptance stage, which represents a defense stage without cognitive bias, self-control is lowest, external control is highest, patients tend to prefer intimate relationships with a few people, and experience the lowest levels of fear and depression.

**Conclusion:**

Three psychological stages of death exist in cancer patients, with differences in proximal and intimacy-related defense mechanisms in each stage. The findings have theoretical and practical implications for psychological interventions for cancer patients.

## 1 Introduction

The latest criteria for definition of death are permanent cessation of brain function” and the corresponding permanent circulatory and neurological cessation (Shemie et al., 2023). Humans have a clear self-awareness of the inevitability of individual death and can generally regulate their own psychological changes autonomously; however, the prospect of cancer-induced death greatly contributes to the physical suffering as well as psychological fear and anxiety of patients with cancer (Greenberg et al., 2014). Cancer is a chronic disease that poses serious threats to human life and quality of life. Some patients view cancer as an indestructible, unpredictable, and unacceptable catastrophe. Although it may not lead to immediate death, the fear of cancer recurrence or metastasis may lead to or exacerbate underlying and “paralyzing” mental disorders such as terror, depression, anxiety, and despair (Sharpe et al., 2018), especially in patients in partial remission (D’Souza et al., 2016). Thus, the threat of cancer-induced death may prompt people to develop defense mechanisms that mitigate this threat and promote psychological and spiritual resilience (Saab et al., 2021). Neuroimaging research has also provided evidence for the association between emotions, such as fear, and defense mechanisms. Recent evidence suggests that the prefrontal cortex (PFC), specifically the medial PFC (mPFC), is responsible for the long-term storage and retrieval of fear memories, while the hippocampus provides contextual information related to learning. The hippocampus projects to the amygdala to encode and transmit contextual representations, ultimately inducing defensive behaviors (Tortora et al., 2023). Defense mechanisms are means of living with death by keeping thoughts about death and the accompanying anxiety in an unconscious state or outside of apparent awareness (Zhou et al., 2018). The proximal defense is the defense mechanism at the conscious level that is responsible for expelling death-related thoughts at the conscious level from consciousness or internalizing them as an acceptable part of consciousness. Numerous studies have also demonstrated that intimacy plays an important role in coping with death-related anxiety and fear. That is, harmonious relationships with others who are mutually influential and dependent on each other can also alleviate death anxiety (Fu and Tang, 2021). Interestingly, cancer promotes the continuous reinforcement, development, and application of proximal and intimacy-related defense mechanisms. Awareness of death enters the conscious level of the cancer patient, triggering the individual’s proximal defenses to expel the death thought outside of consciousness for temporary relief of death anxiety, at which point the initiation of the intimacy defense process can relieve death anxiety at the unconscious level. Similarly, there is theoretical and clinical evidence that the absence or dysfunction of proximal defense mechanisms and the disruption of intimate relationships exacerbate serious consequences such as the development of psychiatric disorders and even suicidal thoughts in cancer patients. Winters (2022) also suggested that psychological changes lead to a disruption of one’s physical and mental balance and predisposition to autoimmune diseases, which can have adverse effects on cancer treatment, recovery, and social stability (Grossman et al., 2018; Courtney et al., 2020). Furthermore, related studies have shown that the organic combination of proximal and intimate relationship defenses have somewhat similar effects to that of palliative care for patients with advanced cancer (Snaman et al., 2020; Gerlach et al., 2022).

Pyszczynski et al. (1999) extended the terror management theory (TMT) framework by proposing a Dual-Process Defense Model that includes proximal and distal defenses. Greenberg et al. (2003) experimentally demonstrated the existence, applicability, and comprehensiveness of distal and proximal defenses. In addition, Kübler-Ross, a pioneering psychologist in the field of death science, proposed the five stages of dying theory based on clinical studies of a large number of cancer patients: denial and isolation, anger, bargaining, depression, and acceptance (Kübler-Ross et al., 1972). Similar to the five-stage theory of dying proposed by Kübler-Ross, Greenberg et al. proposed that proximal defenses defuse or explain the threat of death at different levels of abstraction, which include different degrees of cognitive bias, such as the three levels of defense without cognitive processes, defense with cognitive bias, and defense without cognitive bias.

In a two-year study by Prickett and Timmermans (2022) of a volunteer community in the United States that gathered to bury unclaimed or “abandoned” infants, it was found that people who gathered together transformed potentially negative factors into group-forming events and aligned social experiences, which is somewhat similar to the experience of intimate relationships (Bailey and Walter, 2016; Ådland et al., 2022), which may create a sense of group solidarity and enhance emotional energy, thereby helping cancer patients to mitigate the threat of death. Currently, a large body of research demonstrates that intimate relationships, in addition to having a positive impact on human survivorship and reproductive adaptability (Grebe et al., 2019), play an important role in dealing with death-related anxiety and fear (Xiao et al., 2022). Plusnin et al. (2018) found that several fundamental characteristics of intimate relationships contribute to their defensive roles against fear and anxiety about death. The sense of immortality in intimate relationships can directly alleviate primitive and intense fears, especially the fear of desolation and forgetfulness in the case of dying alone, which helps to reduce the normative perception that “biological death is the same as everything going to zero” (Fairlamb and Juhl, 2022).

By integrating Kübler-Ross’s stages of dying theory and Greenberg et al.’s TMT, it was found that, initially, patients with cancer in the first stage of dying generally exhibit significant denial and avoidance defense mechanisms, and non-cognitive processes at the proximal defense level. The defense mechanisms at this stage are derived from the most primitive self-protective instincts, where people are psychologically and physiologically unwilling to accept the premature end of life, are in an irrational and meaningless state, and desire close intimacy from more people. The stage after denial and avoidance is bargaining, where people facing the threat of death start to change from the initial “turn and run posture” to “turn around and face death.” During this stage, individuals develop logical but biased perceptions; people want to reach some kind of consensus with the outside world, make their own promises, etc., mainly to delay death. At this stage, cancer patients prefer a relationship mode that includes many people but not intimacy.

In this study, the final stage, acceptance of death, is divided into neutral acceptance, escape acceptance, and convergent acceptance. Although all three represent acceptance of death, there is a huge difference in the real level of rationality. Neutral acceptance is a real level of defense that represents a non-cognitive bias. The patient recognizes the fact of his/her inevitable death from the bottom of his/her heart, which represents a real stage of rational thinking about the end of life, that is, a positive step after bargaining failure. At this time, patients with cancer feel that interpersonal relationships with a few people and close relationships are safer and more comfortable. Escape acceptance, on the other hand, is an escape from the reality of suffering, the decision to give up after a failed bargain, and is a negative expression. Convergent acceptance—denying the fact of inevitable death, seeing death as a pathway to a better afterlife, or believing that one will take on some other form of existence after death, and by doing so alleviating the fear and anxiety of death, with religious overtones (Wong et al., 1994). Although psychologists have conducted extensive research on death threats and defense behaviors, researchers have also gained a better understanding of various death defense behaviors. However, currently a large amount of research has focused on imagined mortality salience (MS) and subsequent mortality salience effects, with relatively little research on real mortality salience and subsequent defense effects. This study selects cancer patients as the research object to explore whether there are more distinct psychological stages of dying in cancer patients at the conscious level under real mortality salience, to further explore the differences between proximal defense and intimacy-related defense mechanisms in each stage, and to discuss the potential role of defense mechanisms for mental health interventions in cancer patients.

## 2 Materials and methods

### 2.1 Participants

A total of 220 cancer patients were recruited for this study from inpatients and special outpatients in the oncology department of a tertiary hospital. All participants were aware that they had cancer. All participants volunteered to participate in this study.

### 2.2 Ethical approval and informed consent

This study was approved by the Institutional Review Board of Southwest University and the Affiliated Hospital of Southwest Medical University (reference no. XNYD2017268). The purpose, procedures, and benefits of the study were explained to all participants. Participants were also informed of their right to confidentiality and withdrawal. All participants provided written informed consent, in accordance with the human participants’ guidelines of the institutional ethics committee. After completing the experiments, the participants were offered a gift (one set of bowls valued at approximately 3 USD) and monetary rewards (approximately 16 USD) for their participation.

### 2.3 Overall procedure

**Figure 1:**
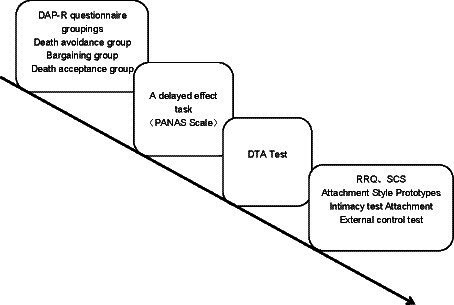
Flow chart of the experiment.

A one-way between-group experimental design was used for the experiment. Upon arrival, the experimenter described the purpose of the study to the participants and explained the reasons for using the relevant measures. A revised version of the Death Attitude Questionnaire was used to divide the subjects into three groups, a death avoidance group, a neutral acceptance group, and a bargaining group. Participants were then asked to complete the Positive and Negative Affect Scale as a delayed effects manipulation task. Subsequently, a self-administered Pinyin version of the Death-Thought Accessibility (DTA) Test was used to measure DTA in cancer patients. Then participants were asked to complete RRQ, SCS, Attachment Style Prototypes, Intimacy test Attachment, and External control tests. Before being tested on the questionnaire, participants were told to answer the questionnaire based on their first response, and then they were given a consent form to read and sign, followed by the questionnaire and a blank envelope. Participants were asked to place the completed questionnaire in the envelope and then place the envelope in a box to indicate that the task had been completed. All participants completed the task at each workstation to ensure their privacy. The flowchart of the experiment is shown in Figure 1.

### 2.4 Measures

The Death Attitude Profile-Revised (DAP-R), prepared by Wong et al. (1994) is a 32-item questionnaire with five dimensions: convergent acceptance of death, fear of death, avoidance of death, escapist acceptance of death, and neutral acceptance of death. All items are scored on a scale of 1–7, with 1 indicating strong agreement and 7 indicating strong disagreement. Participants’ scores on the relevant items were averaged, with lower scores indicating that the participants fit a particular type. The internal consistency and retest reliability of the questionnaire were high (Clements and Rooda, 2000). The main reason for choosing this questionnaire is that it contains three dimensions (avoidance of death, neutral acceptance of death, and fear of death) that are the focus of this experiment: avoidance of death is a first-stage response, neutral acceptance of death is a third-stage response, and fear of death is an emotional response after mortality salience (MS). The only concept missing was the measurement of the bargaining stage, so the researcher devised his own questions for this part, including the following: 1. If God would grant me one wish, I would not regret dying; 2. If I change all my bad habits, it will hopefully make me better; 3. I would give what I have or do more good deeds to prolong my life; 4. I would make a promise to God in exchange for more time; 5. I’ve been thinking about what could be done to extend my lifespan. The dimension of convergent acceptance of death and escapist acceptance of death were removed. In the current study, Cronbach’s α coefficient of the bargaining dimension was 0.86, and those of the other three dimensions of neutral acceptance of death, fear of death, and avoidance of death were 0.76, 0.88, and 0.79, respectively.

The Self-Control Scale (SCS), with 36 questions, was developed by Tangney et al. (2004). The questionnaire was based on an extensive review of published research on self-control processes and failures by Tangney et al. To develop it, a larger set of 93 items was first generated, covering all areas of self-control failure in the review, including thought control, emotion control, impulse control, enforcement of rules, and breaking habits. A 36-item full version and a 13-item short version of the questionnaire were developed, and the full version was used in this study. According to Unger et al. (2016), Tangney’s SCS can be used in China. All items are scored from 1 to 5, with 1 indicating very non-compliant and 5 indicating very compliant; the SCS had high internal consistency, with a Cronbach’s α coefficient of 0.89 and a repeated measures reliability of 0.89.

The Reflection and Rumination Questionnaire (RRQ) consists of two dimensions, rumination and reflection. Rumination refers to recalling and recollecting things related to oneself repeatedly, which is a form of irrational thinking. Reflection refers to rational thinking about the self and things related to oneself. This questionnaire was developed by Trapnell and Campbell (1999). The questionnaire consists of 24 items, all of which are scored on a scale from 1 to 5, with 1 indicating complete disagreement and 5 indicating complete agreement. Twelve of the items are related to rumination (e.g., “I always seem to be thinking about something I said or did recently”) and 12 were related to reflection (e.g., “I like to explore my inner self”). The original questionnaire was translated into Hebrew, then into English, and in this study into Chinese. This questionnaire has been validated by several studies (Taubman-Ben-Ari, 2004). In the present study, Cronbach’s α coefficient was 0.81 for the rumination dimension and 0.79 for the reflection dimension. The participants’ scores on the relevant items were averaged, with higher scores indicating that the participants fit a particular type.

Attachment Style Prototypes: This questionnaire was developed by Hazan and Shaver (1987) and is widely used in China and other countries. The questionnaire comprised four items, each corresponding to an attachment style. Participants are asked to determine which option best describes their feelings in an intimate relationship, through which they can be classified into four types of attachment styles: secure, avoidant, anxious, and fearful.

### 2.5 Experimental procedures

#### 2.5.1 External control test

The Self-Control Questionnaire mainly focuses on one’s control over the self and should also test one’s control over the external world. In their 2003 and 2004 studies, Dechesne and Bandt-Law (2019) pointed out that after MS, an individual’s uncertainty about death rises significantly, and to reduce the threat of death uncertainty, their control rises significantly. Therefore, in the present research, to study control in cancer patients, the investigators used the same experimental procedures as in Dechesne and Bandt-Law (2019) who demonstrated that participants who are subjected to MS show a significant increase in control. The investigators of this study hypothesized that cancer patients at different stages, which are characterized by different defense mechanisms, will show significant differences in the magnitude of their control.

The first instructional statement presented in the experimental procedure was: “Hello, welcome to the Experiment on the Measurement of Cognitive Reactivity in Language Skills. Here are the instructions for the experiment, to guide you through the experiment, and to familiarize you with the procedure. The first thing that will appear on the computer screen is a gaze point “+” (duration 2 s), then the letter “A” or “B” will appear, please press 1 to respond when A appears, and press 2 to respond when B appears. Please respond quickly and accurately. If you are ready, please press Q to enter the quiz; if you do not understand, please consult the experimenter.” In this experiment, no practice trial was included because practice trials would contaminate the formal experiment. In the experiment, the participant was told that the letters A and B appeared randomly, but in fact the two letters appeared in a fixed order: “ABBABAB.” The main purpose of the experiment was to examine whether the participants could find the pattern of the two letters in the shortest time, to increase their response speed for each group of letter stimuli. The entire experiment consisted of 40 trials and the time for each letter was 1 s. The participants could answer within one second of the appearance of the letters, and they did not have to wait until the letters disappeared. The dependent variable is the time participants took to respond to the whole trial of “ABBABAB,” and there were 40 recordings for 40 trials. Participants did not rest during the experiment.

#### 2.5.2 Death-thought accessibility test

In this study, a self-programmed phonetic version of the DTA Test was used to measure the participants’ DTA. We utilized a Pinyin version of the DTA measure in this study. In this instrument, if the Pinyin sound is not marked with a tone, different tones could produce different Chinese characters and meanings. A total of 26 pinyin were included, 13 pinyin that could spell death-related characters, 13 pinyin that could not spell death-related characters at all, and the number of death-related characters spelled out was recorded, with a DTA value ranging from 0 to 13 points, with higher scores indicating a greater threat of death at the unconscious level (Zhou et al., 2022).

This experiment focused on whether there were different levels of DTA at different psychological stages of dying in cancer patients. Therefore, instead of matching the cognitive load task, a delayed effect task was used. The Positive and Negative Affective Scale (PANAS) was used as the operational task for the delayed effect.

#### 2.5.3 Intimacy test

Renkema et al. (2009) proposed that people are known to attribute human features to abstract figures and objects, such as emotions and traits. These features, consequently, have the ability to comfort people, and make them feel safer. Large groups provide safety in numbers, and cohesive groups signal affiliation; these are two constructs that may act as helpful buffers against mortality-induced anxiety. So they predicted and validated that, because groups provide safety and cohesive groups signal affiliation, when reminded of their own mortality, people see figures that resemble large and cohesive groups as being more safe, even when these figures consist of non-human stimuli. And the need to be part of large, cohesive groups is so strong that it may even manifest itself in a preference for abstract geometric representations of such groups.

**Figure 2:**
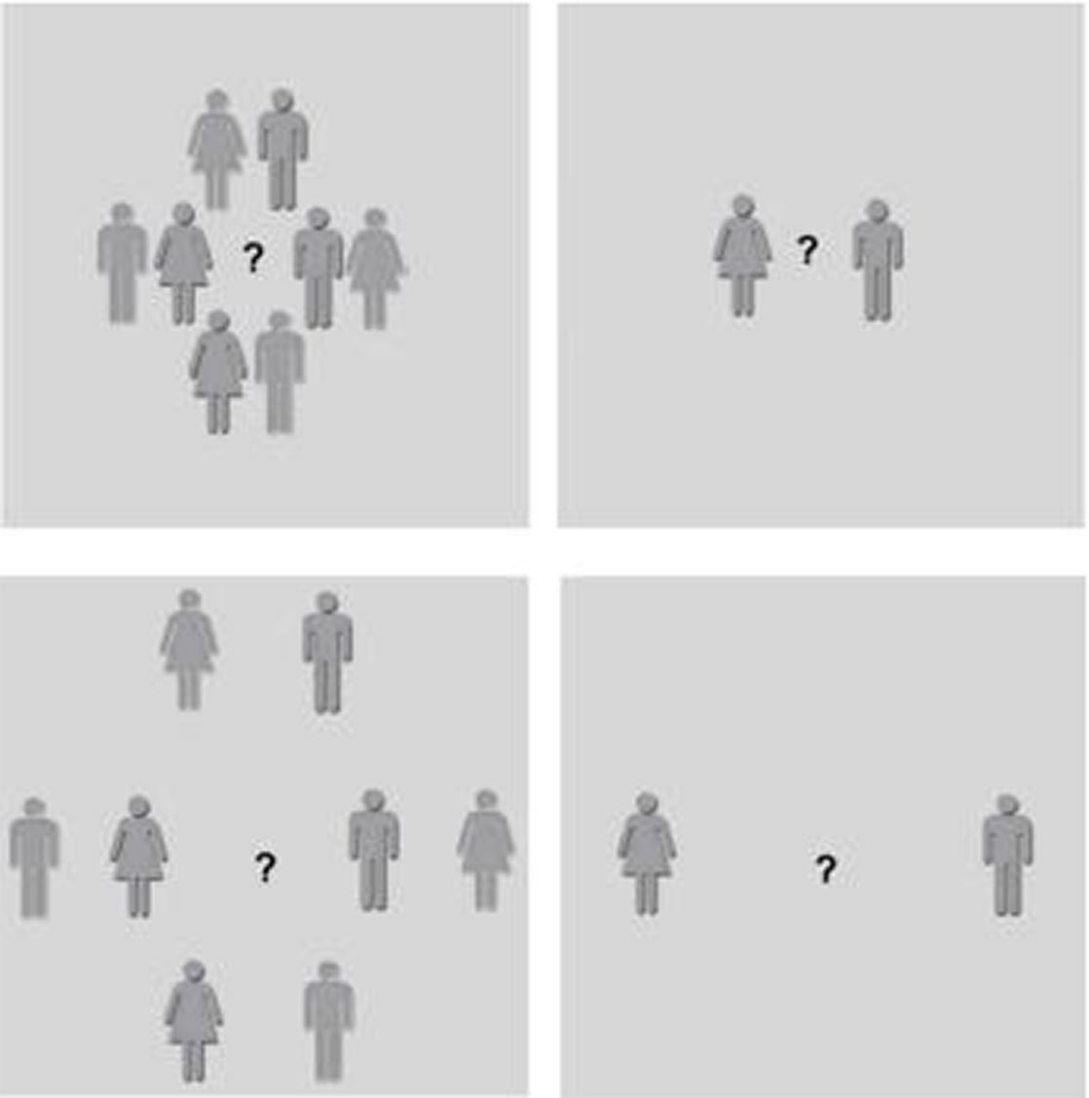
Material I. Participants indicated which picture made them feel safer and more comfortable.

**Figure 3:**
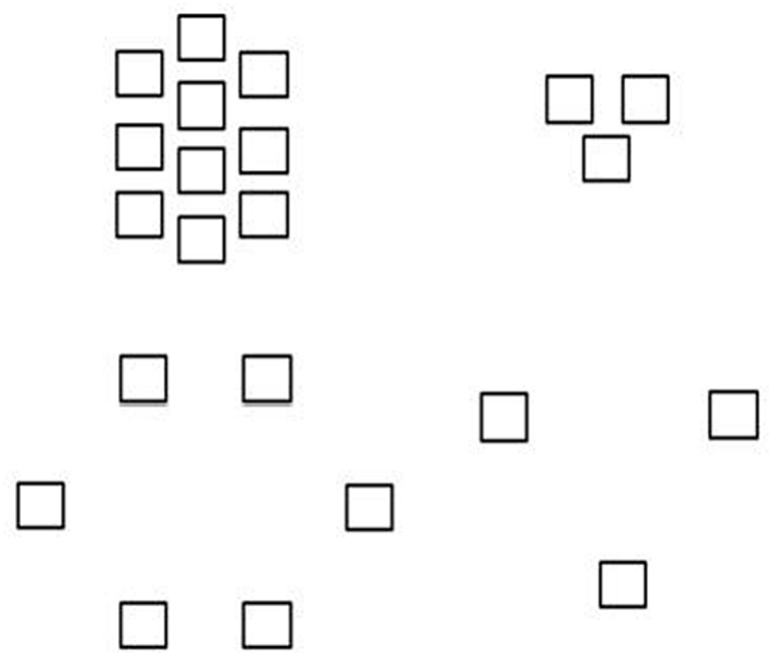
Material II. Geometric squares used as part of the measure of intimacy.

In this study, we used the same experimental materials (Figure 2, material I; Figure 3, material II). Material I consisted of four parsimonious pictures of human figures, with a question mark placed in the middle of the human arrangement in each picture indicating the position of the participant. The number of people in these pictures was either 8 (more) or 2 (less), and the proximity of each person to the question mark was either close or distant. The participants were asked to judge which picture would make them feel safer and more comfortable. For each picture, participants had to choose a score from 1 to 9 (1 = not at all secure; 9 = very much secure). Material II contained square shapes instead of human shapes and consisted of four geometric shapes with sets numbering 10 (more) or 3 (less) and sets representing near or far. The geometric figures did not have the same placement as in material I, but still showed the two effects of more and less and gathered and dispersed. Again, participants were asked to judge the degree to which each picture gave them a sense of security.

These two materials then yielded four states of intimacy: preferring many people and close relationships, preferring few people but close relationships, preferring many people but not close relationships, and preferring few people but not close relationships. The researchers used these two sets of materials to measure which intimacy status cancer patients tended to have at different psychological stages of dying or which level of interpersonal closeness would make them feel comfortable and safe.

## 3 Results

### 3.1 Statistical analysis

Patients were assigned to Death Attitude Profile groups based on the following criteria: participants with mean scores less than or equal to 2 on the death avoidance dimension were included in the death avoidance group; those scoring less than or equal to 2 on the neutral acceptance dimension were included in the neutral acceptance group; and those scoring less than or equal to 2 on the bargaining dimension were included in the bargaining group. Fifty-two participants with scores less than or equal to 2 on two or three simultaneous dimensions were removed. A total of 168 eligible adult patients with cancer participated in this trial, age range 18–75 years, M = 46.28, SD = 12.33, 62.5% female. In the entire group of subjects, the age of the subjects fell mainly in the middle adulthood, with females somewhat outnumbering males, but the data showed that there was no gender difference. There may have been an effect of age in this study, but throughout the data processing we controlled for age by placing it in the covariates. Based on responses to the death attitude questionnaire, patients with cancer were divided into death avoidance (*n* = 54), bargaining (*n* = 59), and death acceptance groups (*n* = 55). The researchers conducted a normality test for each variable, and the Asymp. Sig. (2-tailed) values were all less than 0.05, indicating that the data met the normal distribution. Subsequently, a one-way analysis of variance (ANOVA) was performed for self-control, external control, rumination (irrational thinking), reflection (rational thinking), DTA, and fear of death, followed by two-way comparisons. In the results for intimacy-related defense mechanisms. By excluding subjects with the criterion: intimacy type greater than one, 55 patients who did not fit into a single intimacy type were excluded, and 113 participants were included in the analysis: 31 in the death acceptance group, 48 in the bargaining group, and 34 in the death avoidance group. Each participant rated the four scenarios in the two intimate relationship test images on a scale of 1 to 9. The average score of the same scenario (e.g., close relationships with many people) in both images was taken as the score selected by the participant for that scenario. A multivariate ANOVA was conducted on the four types of intimate relationships in the three groups: close relationships with many people, close relationships with a few people, not close relationships with many people, and not close relationships with a few people, followed by two-way comparisons.

### 3.2 The self-control results

The self-control results for the three groups were *F*(2,165) = 20.19, *p* < 0.001, η^2^ = 0.20. Two-way comparisons using the Bonferroni method showed that self-control in the death avoidance group (M = 180.37, SD = 16.74) was significantly stronger than that in the death acceptance group (M = 163.05, SD = 12.90, *p* < 0.001, 95% CI = [10.67, 23.96]) and bargaining group (M = 169.80, SD = 13.17, *p* < 0.001, 95% CI = [4.04, 17.10]), and self-control was significantly stronger in the bargaining group (M = 169.80, SD = 13.17) than in the death acceptance group (M = 163.05, SD = 12.90, *p* < 0.05, 95% CI = [0.24, 13.24]).

### 3.3 The results of external locus of control

The results of external locus of control for the three groups were *F*(2,165) = 3.85, *p* < 0.05, η^2^ = 0.05. A two-way comparison using the Bonferroni method showed that external locus of control was significantly stronger in the death acceptance group (M = 412.73, SD = 106.14) than in the bargaining group (M = 503.23, SD = 195.13, *p* < 0.05, 95% CI = [−177.38, −3.62]) and marginally significant compared to the death avoidance group (M = 496.43, SD = 247.39, *p* = 0.072, 95% CI = [−172.51, 5.10]), with no significant difference in self-control between the bargaining and death avoidance groups.

### 3.4 The results of rational thinking

The results of rational thinking for the three groups were *F*(2,165) = 12.32, *p* < 0.001, η^2^ = 0.13. A two-by-two comparison using the Bonferroni method showed that the ability to think rationally was significantly stronger in the death acceptance group (M = 43.87, SD = 8.32) than in the death avoidance group (M = 39.22, SD = 4.89, *p* < 0.001, 95% CI = [1.84, 7.47]) and the bargaining group (M = 38.68, SD = 4.32, *p* < 0.001, 95% CI = [2.44, 7.95]), with no significant difference between the bargaining and death avoidance groups.

### 3.5 The results of irrational thinking

The results of irrational thinking for the three groups were *F*(2,165) = 0.82, *p* = 0.443, η^2^ = 0.01. There were no significant differences in the irrational thinking dimension between the death avoidance, death acceptance, and bargaining groups.

### 3.6 The DTA results

The DTA results for the three groups were *F*(2,165) = 13.82, *p* < 0.001, η^2^ = 0.14. A two-by-two comparison using the Bonferroni method showed that the DTA values were significantly higher in the death acceptance group (M = 2.56, SD = 2.64) than in the death avoidance group (M = 0.96, SD = 1.50, *p* < 0.001, 95% CI = [0.68, 2.53]) and bargaining group (M = 0.75, SD = 1.67, *p* < 0.001, 95% CI = [0.91, 2.72]), with no significant difference between the DTA values of the bargaining and death avoidance groups.

### 3.7 The results for fear of death

**Figure 4:**
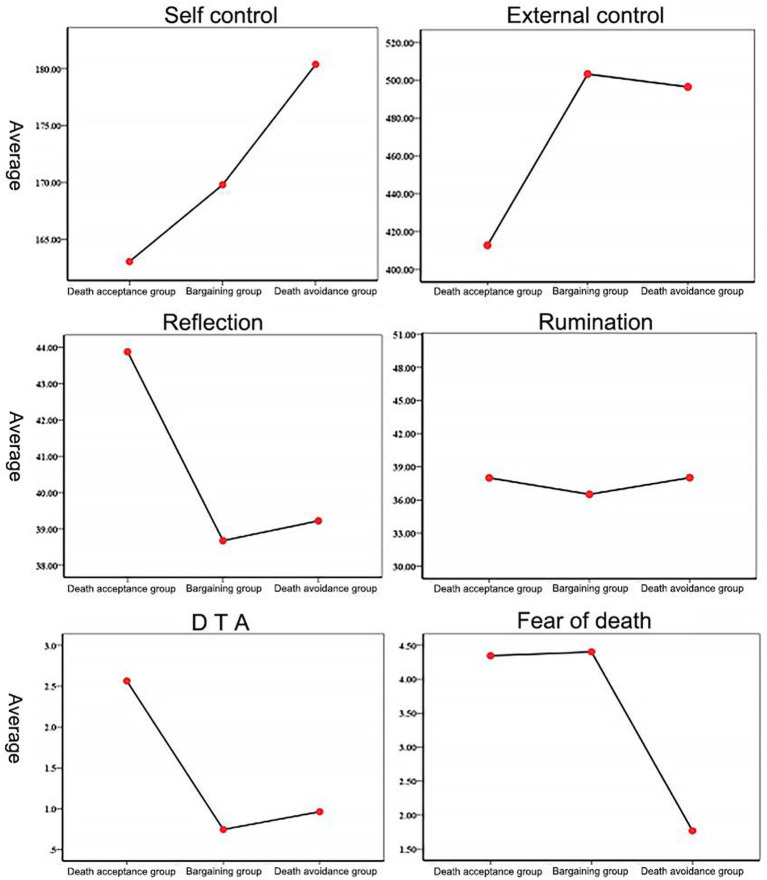
Comparison of the results for control and rational thinking level in the Death Attitude Profile groups.

The results for fear of death in the three groups were *F*(2,165) = 104.40, *p* < 0.001, η^2^ = 0.56. A two-by-two comparison using the Bonferroni method showed that fear of death was significantly stronger in the death avoidance group (M = 1.77, SD = 0.86) than in the death acceptance group (M = 4.35, SD = 1.21, *p* < 0.001, 95% CI = [−3.08, −2.07]) and the bargaining group (M = 4.40, SD = 1.16, *p* < 0.001, 95% CI = [−3.13, −2.14]), with no significant difference between the bargaining and death acceptance groups. Means and standard deviations for all outcomes in the experiment are presented in Table 1 and Figure 4.

**Table 1:** Results for control, rational thinking level, DTA, and fear of death in the Death Attitude Profile groups.

Test items	Death acceptance group *n* = 55		Bargaining group *n* = 59		Death avoidance group *n* = 54	
	M	SD	M	SD	M	SD
Self-control	163.05	12.90	169.80	13.17	180.37	16.74
External control	412.73	106.14	503.23	195.13	496.43	247.39
Reflection	43.87	8.33	38.68	4.32	39.22	4.89
Rumination	38.00	9.37	36.51	5.50	38.02	6.44
DTA	2.56	2.64	0.75	1.67	0.96	1.50
Fear of death	4.35	1.21	4.40	1.16	1.77	0.86

The smaller the value, the stronger the fear of death. The smaller the value of external control, the greater the external control. DTA, death-thought accessibility.

### 3.8 The results for intimacy test

The ANOVA results for the three groups for close relationships with many people were *F*(2,110) = 7.65, *p* < 0.001, η^2^ = 0.13. A two-way comparison of the groups using the Bonferroni method showed that patients in the death avoidance group rated the picture of close relationships with many people significantly higher (M = 7.65, SD = 1.16) than the death acceptance group (M = 5.47, SD = 2.13, *p* < 0.01, 95% CI = [0.67, 3.69]) and bargaining group (M = 5.95, SD = 2.36, *p* < 0.01, 95% CI = [0.33. 3.06]); there was no significant difference between the death acceptance and bargaining groups. The ANOVA results for having fewer close relationships with many people were *F*(2,110) = 5.35, *p* < 0.001, η^2^ = 0.10. A two-way comparison of the groups using the Bonferroni method showed that patients in the bargaining group had significantly higher picture scores for distant relationships (M = 7.19, SD = 1.35) than those in the death acceptance group (M = 5.88. SD = 1.76, *p* < 0.001, 95% CI = [0.51, 2.71]). Picture ratings of distant relationships with multiple people were significantly higher in the death avoidance group (M = 7.03, SD = 1.56) than in the death acceptance group (M = 5.88, SD = 1.76, *p* < 0.01, 95% CI = [0.26, 2.63]). There was no significant difference between the bargaining and death avoidance groups. The ANOVA results for close relationships with fewer people were *F*(2,110) = 6.81, *p* < 0.001, η^2^ = 0.12. A two-by-two comparison of the groups using the Bonferroni method showed that patients in the death avoidance group had significantly higher picture scores for close relationships with fewer people (M = 7.32, SD = 1.53) than those in the bargaining group (M = 5.48, SD = 2.02, *p* < 0.001, 95% CI = [0.61, 3.07]) and death avoidance group (M = 5.82, SD = 1.85, *p* < 0.05, 95% CI = [0.17, 2.83]). There was no significant difference between the bargaining and death avoidance groups. The ANOVA results for less close relationships with few people were *F*(2,110) = 2.75, *p* < 0.05, η^2^ = 0.05. A two-by-two comparison of the groups using the Bonferroni method showed no significant differences. The results for multivariate effect of group were *F*(4,108) = 6.64, *p* < 0.05, η^2^ = 0.11.The means and standard deviations of the above results are shown in Table 2.

**Table 2:** Results for intimacy levels in the Death Attitude Profile groups.

Test items		Cancer patients		
		Death acceptance group *n* = 31	Bargaining group *n* = 48	Death avoidance group *n* = 34
Many people and close relationships	M	5.47	5.95	7.65
	SD	2.13	2.36	1.16
Few people but close relationships	M	7.23	5.48	5.82
	SD	1.53	2.02	1.85
Many people but less close relationships	M	5.58	7.19	7.03
	SD	1.76	1.35	1.56
Few people and less close relationships	M	4.18	3.88	4.57
	SD	2.24	2.11	1.89

### 3.9 The results for attachment type questionnaire

**Figure 5:**
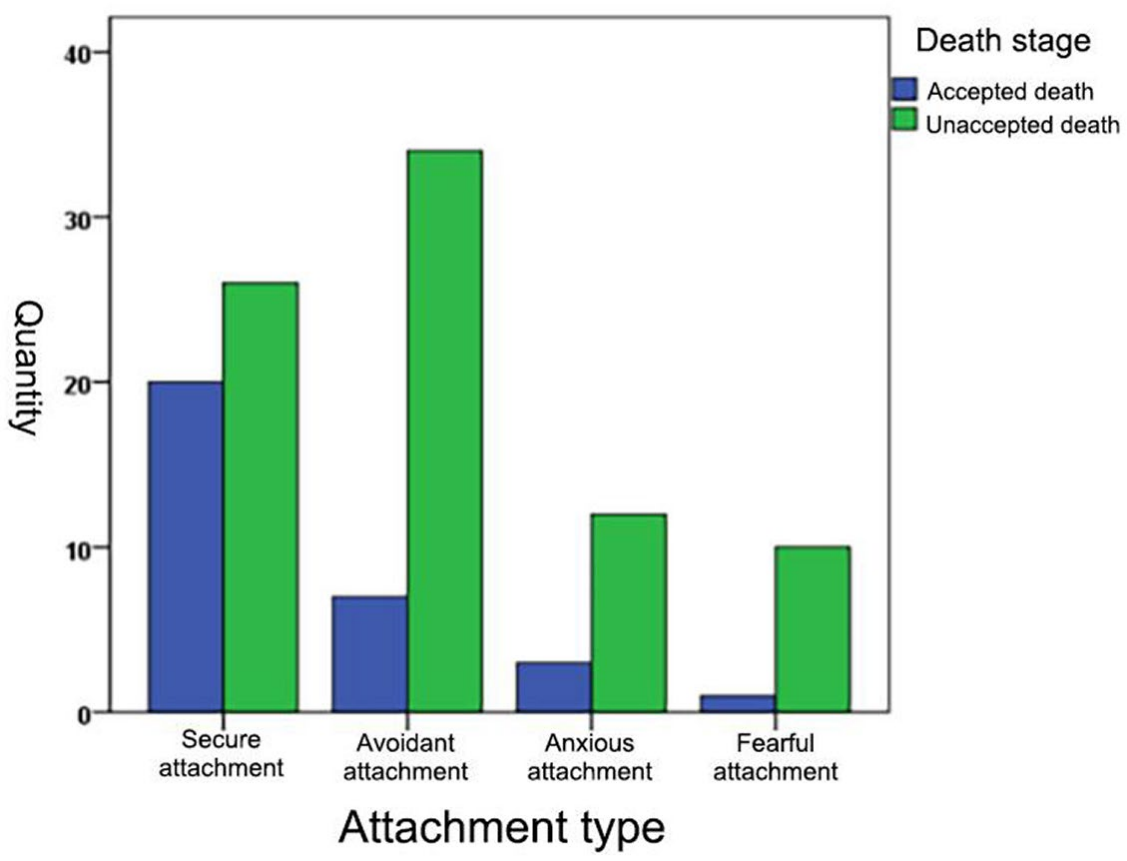
Graph of attachment type versus number of participants who accepted death.

The 113 patients were classified into four attachment types according to the Attachment Type Questionnaire results: 46 cancer patients with secure attachment, 41 with avoidant attachment, 15 with anxious attachment, and 11 with fearful attachment. A chi-square test was performed on the attachment type and the death stage (accepted death and unaccepted death, where the number of people in the bargaining and the avoidance stages were combined) to see if there would be a significant difference in the proportion of people who accepted death among the different attachment types, and the results showed: χ^2^(3) = 10.44, *p* < 0.05. The results are presented in Table 3 and Figure 5.

**Table 3:** Attachment type × stage of death cross tabulation results.

		Death stage		Total
		Accepted	Unaccepted	
Attachment type	Secure attachment	20	26	46
	Avoidant attachment	7	34	41
	Anxious attachment	3	12	15
	Fearful attachment	1	10	11
Total		31	82	113
Percentage		27.4	72.6	

## 4 Discussion

In this experiment, the investigators grouped patients with cancer according to Kübler-Ross’s stages of dying theory and Greenberg et al.’s TMT construct. Three psychological stages of death in cancer patients were measured and differences in proximal and intimacy-related defense mechanisms were explored for each stage. First, patients with cancer in the first stage of avoidance and denial had the highest self-control, after which the level of self-control gradually decreased and was at its lowest in patients in the stage of acceptance of death. In contrast, patients’ level of external control was highest in the stage of accepting death. This result can be explained by the Terror Management Health Model, which suggests that the threat of death increases health behaviors; researchers believe that people are reluctant to accept that they have cancer when they first learn of it. The generation of health behaviors requires strong self-control to maintain, so during the avoidance and denial stages, there is a high level of self-control. At the same time, attention to external things is at its lowest point, so the level of control over the external world is low. However, the prolonged and high-intensity maintenance of health behaviors through self-control consumes various patient resources, and at this time, people enter the bargaining stage; that is, they think they should be rewarded for their efforts. For some patients, however, the results are often disappointing. Repeated negative rewards gradually decrease self-control; conversely, positive rewards can strengthen and maintain self-control. With changes in the psychology of dying, the direction of control changes, shifting from control over oneself to control outside oneself. In the stage of accepting death, control over the self reaches the lowest level. At this time, participants appeared to have increased control over the outside world, for example, they started to think about how to deal with their afterlife. The results of the experiment also showed that the group of cancer patients who accepted death had a significantly higher level of rationality than those in the other two groups. Rational acceptance of death gave the patients peace at the conscious level and significantly decreased their fear. Furthermore, their DTA values after the delay task were significantly higher than those in the bargaining and avoidance denial groups. This suggests that rational acceptance of death reduces the conscious threat of death. The results also showed that in the rumination dimension, the three groups did not show significant differences. This means that although the level of rationality increased in the acceptance of the death group, the irrational factor was still present and did not differ significantly from that in the bargaining and avoidance/denial groups. The coexistence of rationality and irrationality in cancer patients who accept death is justified by the fact that, as Kübler-Ross emphasized, in the third stage, even though the patient is in the shadow of death, he or she still has a glimmer of hope for being cured; that is, the survival instinct still exists while accepting one’s final fate (Kübler-Ross, 1997), which is consistent with human nature. A systematic evaluation showed that defense mechanisms are a dynamic process that can be changed by specific psychological interventions (Di Giuseppe et al., 2018). The results of this study can provide a theoretical basis for psychological interventions for cancer patients, and better explain and predict the process of people’s psychological defense mechanisms after MS. By integrating the contents and processes of the psychology of death, we can gain a comprehensive understanding of the psychological patterns of individuals facing death. This knowledge enables us to design more targeted and effective interventions for physical and mental health. For instance, we can design more tailored group or individual counseling for cancer patients based on their specific psychological characteristics at different stages of death anxiety. Therefore, the findings have significant practical implications.

The findings also revealed significant differences in the need for intimacy among patients with cancer at different psychological stages of dying. Patients in the death avoidance stage craved close intimacy from multiple people, meaning that they would feel safer and more comfortable with more close relationships. This result reaffirms the findings of socioemotional selectivity theory (SST), which suggests that people actively limit their interpersonal connections to emotionally close partners when they perceive time to be strongly limited because emotionally close partners have the greatest potential to provide meaningful social interactions (Fredrickson and Carstensen, 1990; Carstensen and Fredrickson, 1998). However, the study’s findings were even richer in that cancer patients in the death avoidance stage not only adjusted their motivation to be willing to stay with emotionally close people but were also more willing to stay with a larger number of emotionally close people. However, in the bargaining stage, cancer patients preferred interpersonal patterns with more people but at a certain distance from themselves. Researchers believe that this stage is when cancer patients mobilize all their resources to fight against cancer and death and give their maximum psychological commitment to avoiding death; there is a great sense of relaxation and joy if the disease is under control. However, the disease tends to be recurrent, and most patients appear to have a gradual worsening, thus falling into alternating negative emotions such as anger, anxiety, and depression. Thus, patients in the bargaining stage desire to have more people pay attention to them, which will make them feel safe, but do not want to get too close. Researchers believe that overly close interpersonal relationships mean more information, and patients with cancer avoid exposing themselves to more negative news, so they tend to keep a certain distance from people in interpersonal relationships. Patients in the death acceptance stage feel more comfortable and safer with fewer people in close interpersonal relationships. Wanting the closest people to stay close to them narrows the range of emotional choices. Thus, in the stage when one’s time remaining is limited, the study enriches SST by showing that emotional choice undergoes a gradual shift from more intimate relationships with more people to less intimate relationships with fewer people.

This study also explored the relationship between attachment factors, which are strongly associated with intimacy and the stage of dying. The Attachment Type Questionnaire was used to classify patients with cancer into four types, and it was found that a significantly higher number of patients with the secure attachment type entered the acceptance of death stage than those with the other three types. This is because people with secure attachments adopt intimacy as an effective defense mechanism. That is, secure attachment types have positive interpersonal relationships and use intimacy as a defense mechanism, which can be called upon in times of distress, showing that attachment security is associated with enhanced relationship-building and maintenance efforts after MS (Taubman-Ben-Ari et al., 2002; Śmieja et al., 2006; Birnbaum et al., 2011; Cox and Arndt, 2012; Anglin, 2014; Yaakobi et al., 2014). However, patients with the other three non-secure attachment types lack this defense mechanism. For example, anxious types have a strong desire to seek intimacy; however, their overwhelming fear of rejection and low self-confidence usually undermine efforts to achieve intimacy, exposing them to even more anxiety (Mikulincer and Shaver, 2007). Therefore, they are generally less able to feel secure and comfortable through intimacy, and thus find it challenging to use this defense mechanism effectively. Avoidant and fearful insecure individuals (i.e., emotionally distant, distrustful, and compulsively self-reliant) are almost completely isolated from relationship-based terror management mechanisms and will remain in the avoidance of death or bargaining phases for even longer periods. Insecurely attached cancer patients entering the death acceptance stage are more effective at using nonrelational defense mechanisms (i.e., bolstering one’s cultural worldview and self-esteem). The more people they are relationally close to, the more secure and comfortable they feel. Research on intimacy has strong practical implications. Some studies have pointed out that when faced with death anxiety, the use of intimacy-related defenses takes precedence over cultural worldview and self-esteem defenses (Lu et al., 2019). Combined with the results of this study, individuals who have established a secure attachment can invoke intimacy-related defense mechanisms in times of distress and achieve good defense effects to help ward off death anxiety threats. Therefore, the early establishment of attachment has a significant impact on individuals, and it is crucial to pay attention to the development of attachment during individual growth. Additionally, our findings suggest that we can design targeted psychological interventions for cancer patients based on their attachment styles. For example, we can use the safe attachment activation paradigm (Toumbelekis et al., 2021) to develop interventions that alleviate death anxiety in cancer patients. Furthermore, our study found that some individuals may lack intimacy-related defense mechanisms or have insecure intimacy-related defense mechanisms. Therefore, we can explore the roles and effects of other defense mechanisms (e.g., cultural worldview and self-esteem) in different stages of death anxiety in cancer patients. This may be a topic for our future research.

Limitations and outlook of this study. First, the age of the subjects in this study varied considerably, and although covariates were conducted in this study to control for the effect of age on this study, the attitudes toward death may be different between the elderly and the young, and future research could further explore the relationship between age and attitudes toward death by using a specific age group as the subject of the study or designing age into the experimental study. Secondly, this study did not involve the investigation of patients with or without offspring, and future research could also focus on whether there are differences in the defense mechanisms of patients with or without offspring. Longitudinal follow-up studies could also be conducted. This study is mainly a cross-sectional study, and the proximal defense mechanism and intimacy defense mechanism discovered and proposed by the researcher need to be further verified through longitudinal follow-up studies (1 year, 2 years, 3 years, 5 years). Finally, intervention studies can be conducted. The results of the study indicate that not every individual is able to activate the intimacy defense. Individuals may have different defense mechanisms that can be activated, and the amount of defense they can exert after activation may also vary. Therefore, strengthening existing defense mechanisms and cultivating the missing ones may enable individuals to be more resilient when facing the threat of death.

## Data availability statement

The datasets presented in this article are not readily available because the data are not publicly available due to privacy or ethical restrictions. Requests to access the datasets should be directed to JZ, 747756299@qq.com.

## Ethics statement

The studies involving humans were approved by the Institutional Review Board of Southwest University and the Affiliated Hospital of Southwest Medical University (reference no. XNYD2017268). The studies were conducted in accordance with the local legislation and institutional requirements. The participants provided their written informed consent to participate in this study.

## Author contributions

JZ: Conceptualization, Data curation, Investigation, Methodology, Project administration, Resources, Software, Writing – original draft, Writing – review & editing. ML: Conceptualization, Investigation, Methodology, Project administration, Software, Validation, Writing – original draft, Writing – review & editing. JD: Writing – original draft. HS: Investigation, Methodology, Project administration, Resources, Supervision, Validation, Writing – review & editing. MS: Investigation, Project administration, Resources, Supervision, Validation, Writing – review & editing.
